# Where Are They Now? Evolution of a Nurse Anesthesia Training School in Ghana and a Survey of Graduates

**DOI:** 10.3389/fpubh.2017.00078

**Published:** 2017-04-13

**Authors:** Melissa G. Potisek, David M. Hatch, Evans Atito-Narh, Jerry Agudogo, Adeyemi J. Olufolabi, Michael Rieker, Holly A. Muir, Medge D. Owen

**Affiliations:** ^1^Department of Anesthesiology, Wake Forest School of Medicine, Winston-Salem, NC, USA; ^2^Department of Anesthesiology, Ghana Health Service, Ridge Regional Hospital, Accra, Ghana; ^3^Department of Anesthesiology, Duke University School of Medicine, Durham, NC, USA; ^4^School of Nurse Anesthesia, Wake Forest Baptist Health, Winston-Salem, NC, USA

**Keywords:** anesthesia training program, low income country, capacity building, anesthesia workforce

## Abstract

Ghana, like other countries in sub-Saharan African, has limited access to surgery. One contributing factor is the inadequate number of anesthesia providers. To address this need, Kybele, Inc., a US-based non-governmental organization, partnered with the Ghana Health Service to establish the third nurse anesthesia training school (NATS) in Ghana. The school, based at Ridge Regional Hospital (RRH) in Accra, opened in October 2009. This paper describes the evolution of the training program and presents the curriculum. Second, the results of a voluntary survey conducted among the first four classes of graduates (2011–2014) are presented to determine their perceived strengths and gaps in training and to identify employment locations and equipment availability. Seventy-five of 93 graduates (81%) responded to the survey. The graduates reported working in 39 hospitals across 7 of the 10 regions in Ghana. Six providers (8%) worked alone and 16 (21%) were one of only two providers. Fifty-three providers (71%) had no physician anesthesiologist at their facility. Most providers had access to basic anesthesia equipment; however, there was limited access to emergency airway equipment. While most graduates felt that their training had prepared them for their current positions, 21% reported experiencing a patient death during anesthesia. The NATS at RRH has been sustained and most of the graduates are working in Ghana, filling an important void. Quality improvement and continuing education must be emphasized in an effort to reduce surgical morbidity and mortality in Ghana.

## Introduction

Globally, an estimated two billion people have limited or no access to surgery and anesthesia (1, 2). Sub-Saharan Africa (SSA) has 25% of the global disease burden but only 3% of the world’s health care workers (3). Challenges to improving surgical capacity are many, including insufficient equipment and supplies, improper distribution of staff, and migration of health-care workers to more developed countries (3, 4). One report found that half of the countries in SSA lost over 30% of the physicians they trained to migration, leaving a tremendous void (3). The number of anesthesia providers, specifically, is well below what is required to provide safe surgery (4, 5). Many low-income countries (LIC) have between 0.1 and 1.4 anesthesia providers per 100,000 citizens, approximately 1/100th of the ratio found in most developed countries (6, 7).

Ghana is a country in SSA with a population of 24 million, yet in 2007 there were only 20 consultant-level anesthesiologists and 200 nurse anesthetists providing surgical anesthesia for the entire country (8, 9). The lack of providers was due, in part, to the paucity of training programs (10). In 2007, there were only three anesthesia training programs (one for physicians and two for nurse anesthetists). The existing nurse anesthesia training programs, Komfo Anokye Teaching Hospital in Kumasi and 37 Military Hospital in Accra, were longstanding and successful, yet the number of graduates were insufficient for the needs of the population. Both required 3 years of prior nursing experience for admission and were designed as 18-month training programs. Following training, most physician and nurse anesthesia providers were concentrated in urban areas working in small private or large referral and teaching hospitals (11). The smaller district hospitals in Ghana were often unable to provide surgery, a fact which was compounded by the lack of anesthesia providers (4, 11). The large hospitals received a high volume of obstetric and surgical cases and were plagued by delay resulting from too many patients and too few resources. This in turn led to high mortality rates (8, 12, 13).

## Background and Rationale

In 2007, Kybele, Inc., a US-based humanitarian organization, began a 5-year partnership with the Ghana Health Service (GHS) to reduce maternal and newborn mortality (13). Ridge Regional Hospital (RRH) in Accra was selected as the primary target facility, because it was the largest GHS hospital in the country. At the project’s onset, there were three anesthesia physicians and six nurse anesthetists for four operating rooms dedicated to both general and obstetric surgery. The hospital had 6,000 annual deliveries and a 36% cesarean delivery rate. Most cesareans were deemed emergencies (13). Patients for emergency cesarean delivery often waited hours and anesthesia personnel availability was a frequent limitation. Access to emergency obstetric surgery has been cited as an indicator of the overall surgical capacity of a health system (14, 15).

In 2009, leaders of RRH and Kybele opened the third nurse anesthesia school in Ghana. Inter-country collaborations, particularly between academic institutions, have proven effective in building local capacity (4). Collaboration to produce a quality education and adequately trained personnel is paramount to the goal of reducing surgical, maternal, and neonatal morbidity and mortality in LIC. In addition, the overall shortage of physicians, such as anesthesiologists, has led to the need to task shift to less specialized providers to increase manpower (2, 4). This paper will primarily describe the evolution of the nurse anesthesia training program and present the curriculum. The secondary aim was to conduct a survey among graduates to determine their perceptions of the strengths and gaps in training as related to their varied work environments and to examine the retention and location of graduates across Ghana.

## Methods

### Program Implementation

In November 2008, an action plan was developed for the establishment of a nurse anesthesia training school (NATS) at RRH. Key meetings were held with stakeholders including the Greater Accra Regional Director, members of the Health Ministry, the GHS human resource director, and Kybele and RRH leaders. By March 2009, a curriculum was created with input from faculty of the existing Ghanaian nurse anesthesia schools, anesthesia educators from among Kybele leaders, and RRH anesthesia providers. To create the school’s curriculum, the Ghanaian faculty obtained the curriculum used at other local schools in Ghana. The US-based faculty brought curriculum from the Duke and Wake Forest Nurse anesthesia programs and curriculum were compared. The local faculty ultimately determined what their curriculum would be. They then went through the arduous task of getting the curriculum approved by the various local governing authorities. When final approval was given by the Health Ministry to open the school, students were recruited via notifications sent to hospitals throughout the country and advertisements in the print media. On September 9, 2009, the first student applicant was interviewed. From September 9 through September 21, 2009, the Ministry of Health and Education provided training for lecturers. On October 6, 2009, the 18-month nurse anesthesia training program was opened. The admission prerequisites and training duration were modeled after existing programs for continuity.

Kybele volunteers serving as guest faculty included practicing academic anesthesiologists, nurse anesthetists, nurse anesthesia students, obstetric anesthesia fellows, and selected anesthesiology residents in their final year of training. These providers came primarily from the following academic medical centers: Wake Forest University, Duke University, Dalhousie University, Vanderbilt University, the University of Pennsylvania, and Yale University. Kybele volunteers traveled to Ghana in small groups each January, May, and September to support the training program. Volunteers provided 1–2 weeks of didactic, small group and clinically oriented teaching in the operating room and classroom. The clinical teaching by the visiting team covered topics such as preoperative assessment, pharmacology, anesthesia delivery systems, patient monitoring, case scenarios, and subspecialty-specific topics. Kybele members helped in preparing students for examinations, served as oral examiners, and participated in matriculation and graduation ceremonies. The students received regular written, oral, and practical examinations throughout the training period to assess knowledge and practical skills. If the Ghanaian faculty assessed a student as inadequately prepared, they were not allowed to graduate, but were retained for additional training and experience.

### Survey

A 39-question survey was developed for program graduates with input from faculty at RRH and Kybele. The survey gathered information about the graduates’ current work environments, locations of employment, and perceived training preparedness. Questions were designed to assess scope of clinical practice, anesthesia-related mortality, and resource and equipment limitations in the facilities where they worked. Graduates received an e-mail or WhatsApp message detailing the purpose of the survey and were then called by a member of Kybele experienced with the conduct of anesthesia but not previously affiliated with the training program. A short introductory script was used to reiterate the purpose of the 10–15 min voluntary survey, to ensure confidentiality and to obtain verbal consent. Calls were made from May 2014 to September 2015 to graduates of four classes (2011, 2012, 2013, and 2014). At least two attempts were made by phone to contact graduates. If unavailable by phone, graduates attending a September 2015 refresher course in Accra were given a paper survey.

## Results

**Figure 1:**
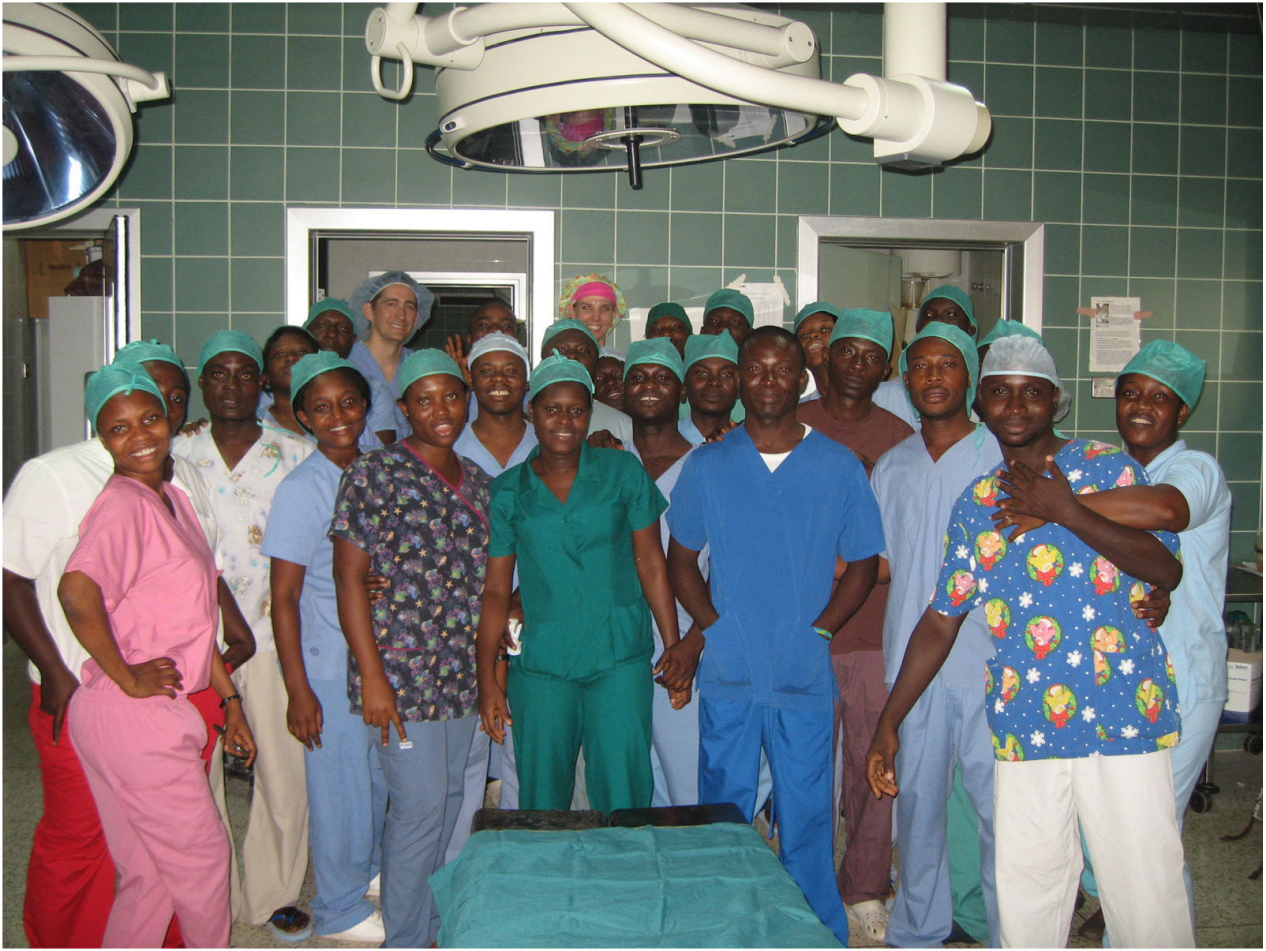
**Nurse anesthesia students at Ridge Regional Hospital with Kybele lecturers**.

The third NATS in Ghana at RRH has enrolled 165 students since 2009. One hundred forty have graduated from the program. The curriculum is presented in Table 1. Forty-seven guest lecturers traveled to Ghana with Kybele from 11 institutions in the USA and Canada from January 2009 through September 2015. Fourteen lectures traveled multiple times to provide educational support for the school (Figure 1). Three educators (one physician and two nurse anesthetists) from RRH were sponsored by Kybele to visit US-based training programs and observe clinical sites of Kybele coordinators.

**Table 1:** **Nurse anesthesia training school curriculum**.

Course title	Total credits
**First semester curriculum**	
Applied anatomy	3
Applied physiology	3
Applied physics and equipment	3
Introduction to research methods and biostatistics	2
Health sociology	2
Applied pharmacology	3
Practicum	3
Total credits	21
**Second semester curriculum**	
Case conference/seminar	**2**
Principles and practice of anesthesia	4
Principles and practice of intensive care	4
Health psychology	2
Project work/research	4
Practicum	3
Total credits	19
**Third semester internship**	
This final part of the program covers a period of 6 months in which the student will work in accordance with his/her work schedule as planned by the facility to which the intern is attached. The intern will ultimately be responsible to the specialist/consultant anesthetist and shall take instructions from him/her. Interns should be responsible for perioperative assessment, intraoperative monitoring of all categories of patients in the theater, postoperative management and monitoring of patients in the recovery ward, postoperative pain management, and the transfer of patients to the wards following surgery. Interns should attend and participate in all clinical presentations in their departments and report back to the school with a written report from the institutions where they underwent internship training	

*Bold indicates different sections/semesters*.

**Figure 2:**
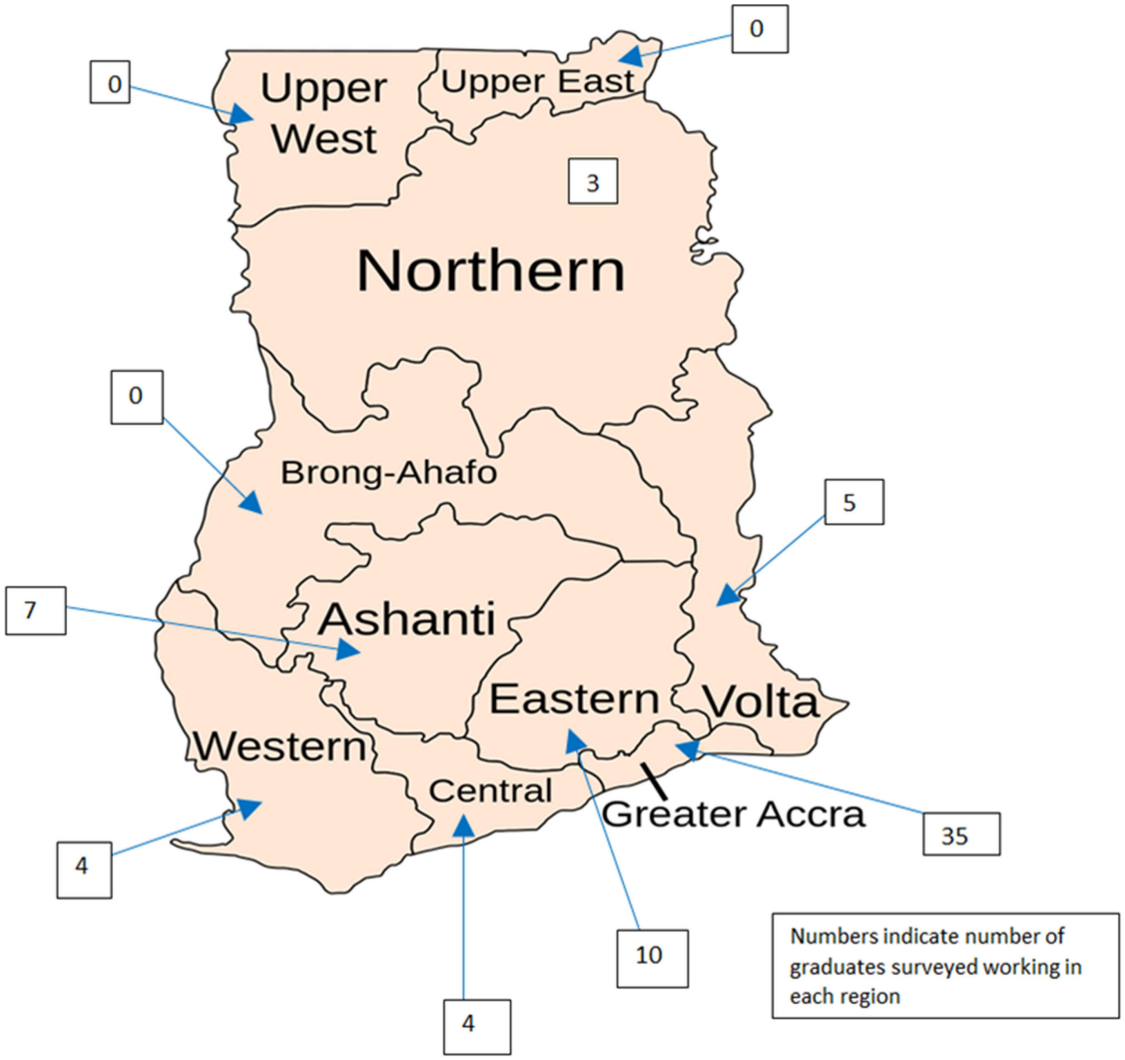
**Map of Ghana with working locations of Nurse Anesthesia Training School graduates**.

Ninety-three students graduating between 2011 and 2014 were surveyed and 75 surveys (81%) were completed. Graduates worked in 39 hospitals across 7 of Ghana’s 10 regions (Figure 2); however, 46% remained in Accra. Regions with no representative graduate included Upper West, Upper East, and Brong-Ahafo (Figure 2). Fifty-three graduates (80%) reported working in government facilities while seven (10%) and four (6%) reported working in private and mission hospitals, respectively. Twenty-three graduates classified their workplace as a regional hospital (34%), 33 reported working in a district hospital (50%), and 3 reported working in a municipal hospital (4%). The average number of nurse anesthetists in a given hospital was 5.4 (range 1–24) with a median of 3. Six providers (8%) reported working alone and 16 (21%) reported working with only one peer. The average number of anesthesiologists per hospital was only 0.4 (range 0–3). Fifty-three providers (71%) reported that no anesthesiologist worked at their facility.

**Figure 3:**
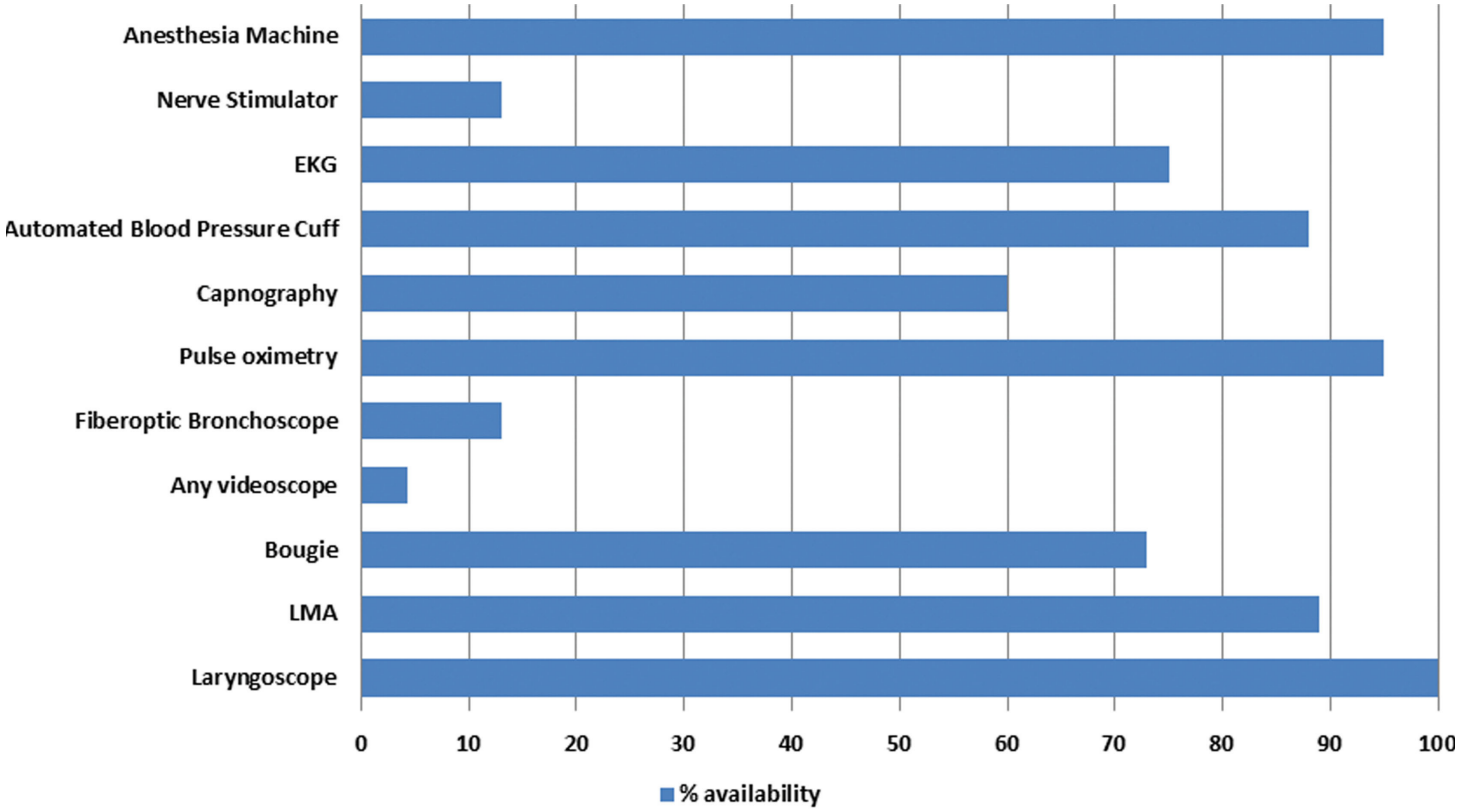
**Equipment availability reported by Nurse Anesthesia Training School graduates**.

Essential equipment availability varied considerably as shown in Figure 3. Notably, 95% of surveyed graduates had access to pulse oximetry and an anesthesia machine but only 58% had capnography. Access to emergency airway equipment was extremely limited, with less than 15% having a videoscope or fiberoptic bronchoscope. Fifteen graduates (21%) had experienced a patient death during anesthesia. Of these deaths, two (13%) were associated with a difficult intubation and five (33%) were within 30 min of spinal anesthesia. The exact cause of the other deaths was unspecified. For cesarean deliveries, 6/61 (10%) reported maternal death during general anesthesia and 10/63 (16%) reported it following spinal anesthesia; however, spinal anesthesia was used more frequently. Graduates reported doing an average of seven cesarean deliveries per month under general anesthesia with a median of six.

**Figure 4:**
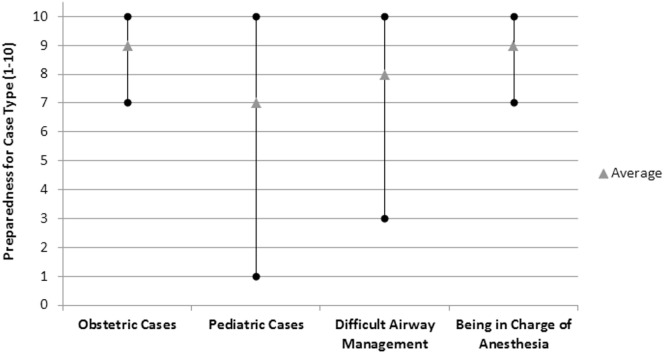
**Quality of training at Ridge Regional Hospital Nurse Anesthesia Training School as perceived by graduates**.

With regard to their perception of preparedness for their current jobs, 64 (85%) felt “definitely” prepared, 8 (11%) felt “somewhat” prepared, 1 (1%) felt “neutral,” 1 (1%) felt “poorly” prepared, and none felt unprepared. Graduates were asked to rate their preparedness to perform specific types of anesthetics on a scale of 1–10 (with 10 being the most prepared). They rated their preparedness highly in all areas, with the highest values assigned to obstetric anesthesia. Responses are displayed in Figure 4.

## Discussion

Anesthesia training programs in LICs should focus on quality, safety, and professionalism while striving for sustainable change that is supported primarily by local resources (10). The development of a locally based NATS to improve capacity is likely to be more cost-effective and viable than reliance on outside resources to meet surgical and healthcare needs. Established as only the third nurse anesthesia school in Ghana, the training program at RRH is addressing a severe shortage of anesthesia providers. The school’s administration is based entirely in Ghana, thus supporting the goal of independence and sustainability. Kybele, as a multinational and interdisciplinary organization, provides mentoring in curriculum design, evidence-based standards, quality improvement, and systems development (13). Kybele volunteers continue to visit for 1–2 weeks at three times per year, serving as guest faculty and supplementing educational topics that are assigned by the Ghanaian administrators. The survey results represent an initiative to evaluate the strengths and weaknesses of the curriculum and to identify barriers in the implementation of safe anesthesia practice in Ghana. The limitations of this survey include incomplete responses, variable interpretation of questions, and recall error, but the results are valuable for program improvement and optimization. It also underscores the importance of partnership in addressing extreme global health anesthesia shortages.

The survey found that over 80% of the first four classes of NATS graduates work in Ghana. This helps fill an important healthcare gap, especially in areas without other providers. The provider retention that the survey demonstrated is encouraging given that health-care emigration has been so problematic in SSA. Furthermore, we found that half of the graduates reported working in district hospitals, which is valuable given that many such facilities have limited medical staff (7). District hospitals, in particular, need adequately trained anesthetists because many procedures in these facilities are emergent and high perioperative mortality rates have been attributed to an insufficient supply and education level of providers (7, 11, 16). Other successful training programs in LICs have developed strategies to retain providers. A NATS in Kenya, for example, has students “sent” to training from rural communities to which they return with a binding work agreement following graduation (17). This was also true for some of the graduates of the NATS in Ghana. Other students were hired by the hospitals to which they were deployed for their internships or posted by the GHS.

The safe provision of anesthesia is limited when essential equipment and monitors are unavailable or poorly functioning. It is reassuring to note that basic equipment availability reported by our graduates is better than what has been shown in other settings (5, 17). For instance, a 2012 survey of 22 LICs reported that only 50% of facilities had an anesthesia machine and pulse oximeter (18). Another report estimated that 40–80% of equipment in LICs is out of service and that 70% of operating rooms in SSA lack pulse oximetry (19). Our survey, conversely, found that 95% of graduates had access to an anesthesia machine and pulse oximeter. The availability of biomedical technical assistance for the repair of broken equipment was not addressed; however, it is likely that equipment maintenance in a tropical climate with frequent electrical fluctuations remains a significant challenge (18). Despite the availability of basic anesthesia equipment, access to emergency airway supplies was variable. While graduates did report almost universal availability of a laryngoscope and nearly 90% availability of the laryngeal mask airway, only 73% had access to a bougie and less than 15% had access to any sort of videoscope. Poor access to proper medication, equipment, and blood products can limit anesthesia practice, compromise patient safety, and can undermine training, thus contributing to provider frustration and job dissatisfaction (6). Kybele has donated emergency airway and other necessary equipment to providers. However, some of the graduates are located remotely and until the survey was conducted, conditions and equipment availability were not known. Other governmental and non-governmental organizations such as Lifebox and Gradian Health Systems are also providing necessary monitoring equipment, ventilators, and anesthesia machines.

It is unclear from this survey if the lack of airway equipment contributed to maternal morbidity or mortality, but the number of deaths reported during anesthesia is troubling. At least 1 maternal death was said to have resulted from a difficult intubation, and 16 other deaths occurred during either spinal or general anesthesia, though the causes are not known. Evaluating safety is beyond the scope of this study. Providing safe care and optimal outcome depends on a multitude of factors, some of which are outside the parameters of anesthesia administration and can include things such as the availability of blood products and emergency medication, electricity, the initial disease state of the patient, timeliness of care, and the availability of backup personnel. The reported number of deaths, however, underscores the need to reinforce safe practice and vigilance while also improving access to emergency equipment and supplies. The risk of anesthesia-related maternal death is estimated to be the highest in SSA (1.5 per 1000) compared to other LICs (19). This highlights the need for better data and root cause analysis to maximize training and quality-improvement efforts (19). Future queries might seek clarification into the specific causes of maternal mortality in the experience of graduates in order to more appropriately allocate resources and direct didactics on sentinel events.

Despite the limitations, most of the surveyed graduates felt prepared for their roles as anesthetists in Ghana. There are multiple ways to access preparedness after training, such as oral or written examinations, faculty appraisal, and student self-evaluation. These are utilized in well-resourced training programs and were also utilized in Ghana. The inter-cultural aspect of this program is valuable because it exposed the students to a broader range of topics they might not have otherwise learned about. Given the different hospital settings the graduates would be practicing in, we felt a personalized self-assessment was a valuable starting point to assess their perception of training preparedness as it applied to their new work environments. Furthermore, their sense of preparedness might also have been, in part, due to the selection criteria for admission which requires nursing training and 3 years of work experience (20). It is concerning, however, that many providers reported working alone without an anesthesiologist or other colleagues for support. The lack of appropriate collaboration is a contributing factor to the inconsistent quality of care that has been documented in SSA (5, 12). The NATS went through a rigorous approval process by the Health Ministry and policies in Ghana do allow nurse anesthetists from approved programs to practice independently.

However, having the support of colleagues for problem solving, technical assistance, and capacity building is a goal for many hospitals in Ghana and throughout SSA and could increase job satisfaction and the quality of care (21).

Another strategy to offset provider isolation is continuing education and support. Nurse anesthetists worldwide have called for better access to continuing education in order to improve their skills, knowledge, and practice (22). The International Federation of Nurse Anesthetists has recently published a model curriculum for an 18- to 24-month certificate (non-degree) program as well as applications for program recognition and accreditation (23). In addition, the website (www.ifna.site) contains a wealth of teaching material, practice standards, and guidelines available for download. Health Volunteers Overseas has developed an on-line curriculum that could be a useful adjunct to graduates in the future (24). In addition, we hope to continue a recently debuted annual refresher course for continuing education attended by 74 NATS graduates and maintain regular communication. Future refresher topics should include pediatric anesthesia and difficult airway management. Based on the survey’s results, the school’s curriculum has been changed to include emergency management, including advanced airway management techniques and cardiopulmonary resuscitation. Furthermore, the school recently expanded the class size from 25 to 38 and the length of training from 18 to 24 months while also advancing from a diploma to a degree program that is being supported by the University of Cape Coast. In addition, practicing nurse anesthetists now have the opportunity to return to school for 6 months to “top-up” their previous training and receive their degrees. Hopefully, this will enhance the clout of nurse anesthetists in Ghana to further improve job satisfaction and provider retention (10).

There are few publications regarding the outcomes of global health collaborations to increase the number of anesthesia providers in LICs, though various organizations are working in this capacity (10, 17). Notably, work is being done by the American Society of Anesthesiologists, the Canadian Society of Anaesthesia, and the World Federation Societies of Anesthesiology to increase physician anesthesia provision and by the International Federation of Nurse Anesthetists to guide nurse anesthesia program development. We hope that by sharing our experience with establishing the third NATS in Ghana, others can refine or develop their own partnerships to increase the capacity of anesthesia care. NATS is a success in that it has been entirely locally sustained and is expanding based on its success. We aim to continue following graduates of the NATS, working with the local administration to improve the curriculum, the capacity to provide quality patient care, and to ultimately reduce surgical morbidity and mortality in Ghana.

## Conclusion

A global health partnership recognized a gap in the provision of anesthesia care in Ghana. Through joint collaboration, the third NATS in Ghana was established at RRH in Accra. The school has been locally sustained and most of the graduates are working in Ghana, filling an important void. Quality improvement and continuing education must be ongoing in an effort to maximize surgical safety in Ghana.

## Ethics Statement

Institutional approval to conduct the survey was granted by Wake Forest University Health Sciences and the Ghana Health Service.

## Author Contributions

MP analyzed and interpreted the survey results and drafted the manuscript; DH co-designed and oversaw survey execution and assisted with data analysis and interpretation; EA-N developed and executed the curriculum, and co-designed the survey; JA developed and executed the curriculum and co-designed the survey; AO co-designed and assisted with survey execution, coordinated and oversaw lecturers, and developed educational content; MR co-developed the curriculum and co-designed the survey; HM co-developed the curriculum, coordinated lecturers; MO co-designed the survey, analyzed, and interpreted the survey results and drafted the manuscript. All authors have reviewed the manuscript and are accountable to the accuracy and integrity of this work. All authors give final approval for publication.

## Conflict of Interest Statement

The authors declare that the research was conducted in the absence of any commercial or financial relationships that could be construed as a potential conflict of interest.
